# Differences in influencing mechanism of clinicians’ adoption behavior for liver cancer screening technology between the leading and subordinate hospitals within medical consortiums

**DOI:** 10.1186/s12885-024-12281-y

**Published:** 2024-04-23

**Authors:** Shiyin Wu, Yuhang Zheng, Lingjie Wang, Wenbin Liu

**Affiliations:** Department of Social Medicine and Health Management, School of Health Management, Fujian Medical University, 1 Xuefubei Road, Minhou District, 350122 Fuzhou, China

**Keywords:** Technology adoption, Theory of planned behavior, Liver cancer screening, Medical consortium, Clinicians

## Abstract

**Background:**

Medical consortiums have been extensively established to facilitate the integration of health resources and bridge the technical gap among member institutions. However, some commonly appropriate technologies remain stagnant in subordinate hospitals, although they have been routinely applied in leading hospitals. Besides, the mechanism underlying differences in clinicians’ adoption behavior at different levels of institutions was unknown. Therefore, this study aimed to investigate the differences in influencing mechanisms of clinicians’ hepatic contrast-enhanced ultrasound technology (CEUS) utilization behavior between leading and subordinate hospitals within medical consortiums, thus providing clues for expanding effective and appropriate technologies within integrated care systems.

**Methods:**

A self-designed scale was developed based on the theory of planned behavior (TPB). A multistage sampling method was applied to investigate clinicians who were aware of CEUS and worked in liver disease-related departments within the sampled medical institutions. The final sample size was 289. AMOS 24.0 software was used to construct multi-group structural equation modeling (SEM) to validate the hypotheses and determine the mechanism of hepatic CEUS utilization.

**Results:**

It revealed that behavioral intention significantly influenced adoption behavior, regardless of whether it was in leading hospitals or subordinate hospitals (*β* = 0.283, *p* < 0.001). Furthermore, behavioral attitude (*β* = 0.361, *p* < 0.001) and perceived behavioral control (*β* = 0.582, *p* < 0.001) exerted significant effects on adoption behavior through behavioral intention. However, in leading hospitals, subjective norm had a significant positive effect on behavioral intention (*β* = 0.183, *p* < 0.01), while it had a significant negative impact on behavioral intention in the subordinate hospitals (*β* = -0.348, *p* < 0.01).

**Conclusion:**

To effectively translate the adoption intention into actual behavior, it is recommended to elucidate the demand and facilitators involved in the process of health technology adoption across leading and subordinate hospitals. Additionally, bolstering technical support and knowledge dissemination within subordinate hospitals while harnessing the influential role of key individuals can further enhance this transformative process.

**Supplementary Information:**

The online version contains supplementary material available at 10.1186/s12885-024-12281-y.

## Background

Medical consortium refers to the medical alliance created by integrating medical resources from the same region with the goal of providing continuous care to patients and improving the efficacy of health care delivery [1, 2]. The establishment of medical consortiums strengthens the communication between the leading hospitals (i.e., government-appointed units responsible for forming the consortium) and subordinate hospitals (i.e., all hospitals within the medical consortium except the lead units), facilitating technology diffusion and utilization within the medical consortium while bridging technical gaps.

However, practical challenges persist within the medical consortium. Some commonly high-quality and appropriate health technologies have been routinely applied in leading hospitals but are far from widespread in subordinate hospitals. Contrast-enhanced ultrasound (CEUS) for the liver, for example, has the advantages of lower cost, high specificity, and sensitivity for early liver cancer screening [3–5] and has been confirmed appropriate at different levels of hospitals and has been routinely applied in leading hospitals. Its implementation remains stagnant in qualified subordinate hospitals [6]. This fact reminds us that some differences exist between leading and subordinate hospitals in influencing mechanism of certain technology adoption [7, 8]. To effectively facilitate health technology diffusion and utilization, it is imperative to investigate the differences in the influencing mechanisms underlying this process.

Researchers have been trying to use various theories to explain the rational mechanisms of human behaviors, such as the theory of planned behavior (TPB) [9], which is one of the most influential and widely used theories to predict behavioral intentions [10]. According to the TPB, intention is a potential motivation for individual behavior and is determined by behavioral attitudes (BA), subjective norm (SN), and perceived behavioral control (PBC). Many research findings [11–15] have shown that TPB has a more accurately defined structure and more substantial explanatory power than many other psychological theories or models. It considers the influence of social pressure and perceived behavioral control on individual behavioral intentions, strengthening the model’s explanatory power [16, 17]. Moreover, it has been applied to interpret specific behaviors of healthcare workers, such as compliance with guidelines, utilization of health technologies, etc [18–22].

Previous studies regarding clinicians’ technology adoption behaviors have mainly been conducted within a single hospital or same-level institutions [23–27], and few studies have investigated the differences in the influencing mechanisms of clinicians’ technology utilization across different level institutions within the medical consortiums. Different groups may exhibit different adoption behaviors towards particular technologies. Nevertheless, it is essential to note that there is some technology exchange and information communication between leading and subordinate hospitals within medical consortiums [28–30], which is not equivalent to two utterly independent medical institutions. Furthermore, since some appropriate health technologies that should be widely promoted have only been applied in leading hospitals but are far from widespread in subordinate hospitals [6], it is necessary to investigate the differences in influencing mechanisms of clinicians’ technology utilization behavior across different levels of hospitals. There is a research gap on the diffusion of health technologies in integrated health systems, which prevents the diffusion of some commonly confirmed high-quality and appropriate health technologies across different levels of hospitals.

Therefore, given the necessary diffusion of appropriate health technologies within medical consortiums, coupled with the paucity of studies targeting differences in influencing mechanisms of clinicians’ technology utilization at different levels of institutions, this study aimed to employ TPB and multi-group SEM to identify differences in influencing mechanisms of clinicians’ adoption behavior for liver cancer screening technology between leading and subordinate hospitals within the medical consortium. The findings of this study will not only directly contribute to the expansion of hepatic CEUS use among different member institutions but also provide valuable references for promoting the diffusion and utilization of other effective and appropriate technology.

## Methods

### Theoretical model and hypotheses

**Fig. 1:**
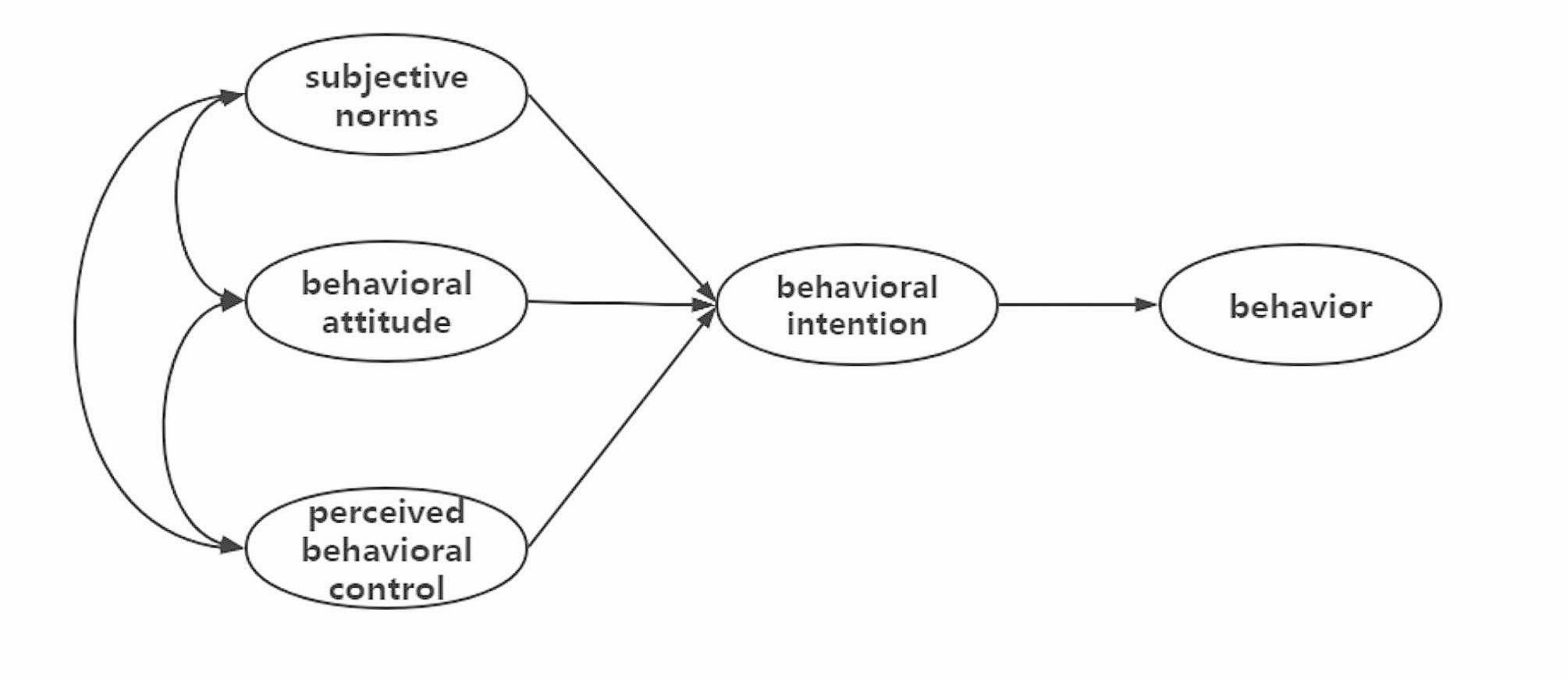
A framework for a theoretical model of planned behavior

To investigate the mechanism of CEUS utilization by clinicians and to compare the differences in influencing paths between leading and subordinate hospitals within the medical consortium, this study was based on the theory of TPB and incorporated five key elements: behavioral attitude, subjective norm, perceived behavioral control, behavioral intention, and final utilization behavior. BA encompasses an individual’s comprehensive evaluations of behavior, including both positive and negative assessments of specific actions. SN refers to the social influence individuals experience when deciding whether or not to engage in a specific behavior, emanating from influential figures such as superiors and colleagues. PBC refers to the perceived level of control individuals have over their actions, considering both facilitators and barriers. The proposed theoretical model is presented in Fig. 1, and the corresponding hypothesis (H) is as follows:

H1a: Clinicians’ utilization behavior is influenced by their intentions to use CEUS.

H1b: Clinicians’ intentions to use CEUS are influenced by their attitude, subjective norm, and perceived behavioral control.

H2: The mechanism influencing the adoption of CEUS techniques by clinicians may differ by hospital level (leading and subordinate hospitals).

### Study design and data collection

A cross-sectional study was conducted from February to August 2019. Given the vastness of China, the different incidence rates of liver cancer in each province, its diverse types of medical consortiums, and the need to maintain a concentrated sample, this study used a multistage sampling method. First, one province was randomly selected from each of the high and low liver cancer prevalence regions in China. In this stage, the provinces of Fujian and Jiangxi were selected on behalf of the high and low hepatic cell carcinoma incidence regions of China, respectively (the incidence of liver cancer in Fujian and Jiangxi were 31.7/100,000 and 23.8/100,000, respectively [31, 32]). Second, all medical consortiums in each province were listed as the sample frame, and two medical consortiums were randomly selected from each province. In this stage, four medical consortiums were selected. Third, a sampling frame of all hospitals in the four medical consortiums was established, and 50% of the hospitals within each healthcare consortium were randomly selected for inclusion in the survey. In this stage, 4–8 hospitals from each medical consortium were selected. Finally, clinicians who were aware of CEUS technology and worked in liver disease-related departments (including hepatology, hepatobiliary surgery, oncology, gastroenterology, etc.) were invited to participate. Trained facilitators accompanied the distribution of questionnaires in collaboration with sampled hospitals, ensuring that participants comprehended the study’s objectives and data utilization. Voluntary and anonymous participation was ensured, and informed consent was obtained from all individuals involved.

According to the formula for estimating population proportion *n* = [DEFA*Np (1 − p)] **/** [d^2^ /Z^2^_1−α/2_ *(N-1) + p* (1-p)] [33]. DEFA is the design effect. Z = 1.96. α = 0.05. The total population (N) of medical personnel in the Fujian and Jiangxi Provinces is 188,297 (*N* = 188,297). The expected proportion (p) of clinicians who were aware of CEUS and worked in liver disease-related departments was 50% (*p* = 0.50), with confidence limits of 10% (d = 0.1). Since this study utilized a multistage sampling method, the design effect was set at 2. The sample size calculation also considered a non-response rate of 10.0%. Therefore, the minimum sample size required was 211. This study ultimately included 289 samples, which could meet the required sample size. The sampling flow chart can be found in Additional File 1.

### Questionnaire

According to the theoretical framework depicted in Fig. 1 and the scale content devised by previous studies, a meticulously structured questionnaire with 21 items was developed. The questionnaire encompasses three distinct sections:

The first section encompassed the fundamental particulars of participants, including six socio-demographic variables, such as gender, age, education level, professional title, years in practice, and level of the medical institution. The age was classified into three groups: <35, 35 ∼ 44, and > 45 years old. Education level was divided into junior college or below, bachelor, master’s degree or above. The professional title of clinicians included three levels: junior, intermediate, and senior. Years in practice were classified into five groups: <5, 5 ∼ 10, 11 ∼ 20, 21 ∼ 30, and > 30 years. The level of the medical institution included leading hospitals and subordinate hospitals.

The second part aimed to assess the clinicians’ utilization of CEUS techniques in the past year (e.g., over the past year, the likelihood of ordering a hepatic CEUS on all working days when appropriate clinical situations arise). The dimension was measured by three items, and each item was evaluated on a six-point Likert scale ranging from 0 (never) to 5 (very high). High scores indicated a high level of hepatic CEUS adoption behavior (e.g., higher frequency of conducting hepatic CEUS reflects a higher level of CEUS behavioral intentions).

The third part was to measure clinicians’ perceptions toward CEUS use, including 12 items from four dimensions, namely behavior intention, attitude, subjective norm, and perceived behavioral control. “Behavior intention” assesses clinicians’ willingness to use CEUS in the diagnosis of early liver cancer (e.g., if there is an opportunity, I would like to apply contrast-enhanced ultrasound to the early diagnosis of liver cancer). “Behavior attitude” was mainly measured from the perspective of whether the use of CEUS technology was consistent with the clinicians’ personal values (e.g., I think it’s a right thing to use CEUS for early diagnosis of liver cancer). “Subjective norm” focuses on measuring the perceptions of CEUS by those who are essential to the clinicians (e.g., people who are important to me tend to use CEUS for early diagnosis of liver cancer). “Perceived behavior control” measures the benefit of CEUS in clinicians’ liver cancer diagnosis (e.g., using CEUS can give me more choices in diagnosing liver cancer). Each of the dimensions was measured by three items, which were derived from the TPB scale and restated to fit the practical context of CEUS. All the 12 items were measured by using a 5-point Likert scale ranging from 1 (strongly disagree) to 5 (strongly agree). More details about the questionnaire can be found in Additional File 2.

### Reliability and validity

Six experts were invited to assess the importance and appropriateness of the questionnaire items. Based on their feedback, minor modifications were made to the questionnaire items to enhance its clarity. Subsequently, a pilot test was conducted on a convenient and representative sample of 30–40 subjects using the revised questionnaire. Following the pilot test, additional modifications were made, including changes to clarify sentence structure. Cronbach’s alpha was used to determine the reliability of each measurement item and the whole questionnaire, and it was reported to be greater than the recommended threshold of 0.7, indicating that internal consistency could be considered adequate. Regarding the validity, the results showed that the Kaiser-Meyer-Olkin (KMO) value of 0.929 and Bartlett’s test of sphericity was significant, indicating the great suitability of this instrument for validity estimate. As the three commonly used indicators to assess convergent validity, namely factor loading of each item, average variance extracted (AVE), and composite reliability (CR), the results showed the value of these three indicators were above the recommended value of 0.5 [34], 0.5 [35], and 0.7 [36], respectively, which indicates an acceptable convergent validity. More details can be found in Table A of Additional File 3. The results of the exploratory factor analysis showed that the five factors had a cumulative variance explained of 85.24%. Varimax rotation was used to further define the included factors. Almost all items in each dimension were loaded with five different factors, which fit well with the proposed framework and indicated that the validity of the questionnaire was acceptable. Factors 1 to 5 explained 18.76%, 17.96%, 18.04%, 13.93%, and 16.55% of the total variance, respectively. More details can be found in Tables B1 and B2 of Additional File 3.

### Common method bias tests

Since this study used scales for measurement, there may be an issue of common method bias. Podsakoff et al. [37] suggested using the controlling for the effect of an unmeasured latent method factor (ULMC) to assess common method bias. Mplus 8.3 was used to test ULMC, and the results showed that ∆RMSEA = 0.008 < 0.05, ∆SRMR = 0.006 < 0.05, ∆CFI = 0.009 < 0.1, ∆TLI = 0.007 < 0.1, indicating the degree of model fit did not significantly improve after adding the common method factor, so there was no significant common method bias in this study.

### Data analysis

In this study, SPSS 26.0 and AMOS 24.0 software programs were the two main statistical tools used to analyze the data. Firstly, late respondents were used as proxies for non-respondents to assess potential non-response bias. Specifically, early respondents and late respondents were compared in terms of gender, age, education level, behavior score, behavioral intention score, etc. Secondly, the study assessed the reliability and validity through Cronbach’s alpha coefficient and exploratory factor analysis to tell whether the questionnaire was acceptable. Thirdly, descriptive statistics were performed to illustrate the demographic characteristics of the participants, and independent *t*-tests were used to compare the scores of each factor in leading and subordinate hospitals, respectively. Finally, multi-group SEM was conducted to validate the hypotheses and determine the mechanism of hepatic CEUS diffusion and utilization within leading and subordinate hospitals of the medical consortium. Chi-square/df, comparative fit index (CFI), Tucker-Lewis Index (TLI), root mean square error of approximation (RMSEA), and standardized root mean square residual (SRMR) were used to evaluate the model fit. The criteria commonly used are: (1) Chi-square/df < 5; (2) CFI > 0.9; (3) TLI > 0.9; (4) RMSEA < 0.08; (5) SRMR < 0.08. In all analyses, *P* values less than 0.05 were considered significant.

## Results

### Participant demographics

The demographic characteristics of the 289 participants are presented in Table 1. In this study, the proportion of male (65.1%) participants was higher than female (34.9%). Approximately half of them were 35 years and older (49.5%), and more than 90% had a bachelor’s degree or above. With respect to professional titles, about two-fifths had intermediate grades (40.1%), while senior and junior titles accounted for 23.6% and 36.3%, respectively. Approximately one third of the participants reported that they had 5–10 years in practice. Besides, nearly half of them worked in the leading hospital (49.1%). Each item for early respondents was similar to those of late respondents, and more details can be found in Additional File 4.

**Table 1:** Demographic characteristics of the 289 participants

Variables	Categories	Frequency (n)	Percentage (%)
Gender	Male	188	65.1
	Female	101	34.9
Age	< 35 years old	146	50.5
	35 ∼ 44 years old	104	36.0
	> 45 years old	39	13.5
Education level	Junior college or below	18	6.2
	Bachelor	154	53.3
	Master or above	117	40.5
Professional title	Junior	105	36.3
	Intermediate	116	40.1
	Senior	68	23.6
Years in practice	<5 years	72	24.9
	5 ∼ 10 years	93	32.2
	11 ∼ 20 years	88	30.4
	21 ∼ 30 years	30	10.4
	>30 years	6	2.1
Level of the medical institution	Leading hospital	142	49.1
	Subordinate hospital	147	50.9

### Measurement scores of CEUS utilization behavior and regarding predictors

Table 2 demonstrates the score of clinicians’ CEUS utilization behavior and regarding predictors within the leading and subordinate hospitals. Overall, the scores of clinicians’ CEUS adoption behavior were low. The mean scores for respondents from leading and subordinate hospitals regarding CEUS adoption behavior were 1.972 and 1.660, respectively. The *t*-test results showed that the CEUS adoption behavior scores of clinicians in leading hospitals were higher than those in subordinate hospitals, while the scores of clinicians’ CEUS behavior intention and perceived behavior control in subordinate hospitals were higher than those in leading hospitals (*P* < 0.05). However, the two groups had no significant differences in the scores of regarding subjective norm and behavioral attitude (*P* > 0.05).

**Table 2:** Measurement score of clinicians’ CEUS utilization behavior and regarding predictors in leading and subordinate hospitals

Variables	Leading hospital	Subordinate hospital	t	P
Behavior	1.972	1.660	2.199	0.029
Behavioral intention	4.113	4.376	-2.730	0.007
Subjective norm	4.160	4.245	-0.875	0.382
Behavioral attitude	3.951	4.068	-1.049	0.295
Perceived behavioral control	4.106	4.297	-2.035	0.043

### Multi-group analysis

**Fig. 2:**
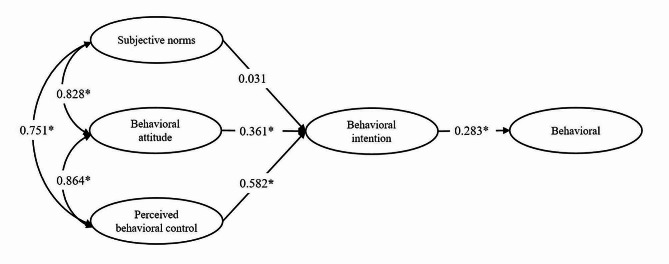
Model of utilization behavior of CEUS among 289 clinicians (*P < 0.001)

**Fig. 3:**
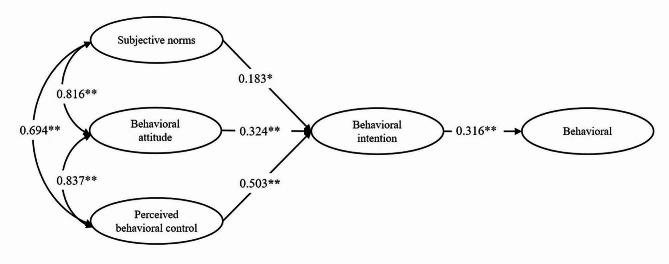
Model of utilization behavior of CEUS among clinicians in leading hospitals. (*P < 0.01, **P < 0.001)

**Fig. 4:**
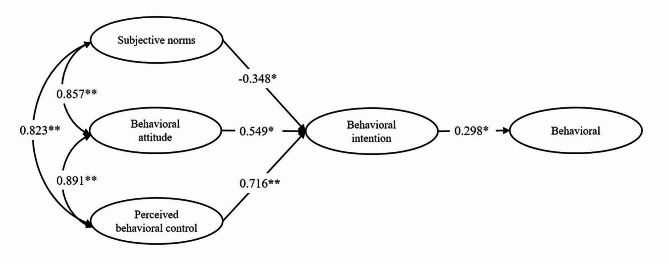
Model of utilization behavior of CEUS among clinicians in subordinate hospitals. (*P < 0.01, **P < 0.001)

Based on TPB theory, multi-group SEM was performed to determine the direct and indirect relationships between SN, PBC, and BA on clinicians’ technology adoption behavior in both leading hospitals and subordinate hospitals. A reference model calculated with the full sample showed the goodness of fit, meeting the criteria for specified values (*χ*^*2*^/*df* = 2.538, *CFI* = 0.972, *TLI* = 0.964, and *RMSEA* = 0.073). The SEM for TPB showed a significant correlation between SN, BA, and PBC (*P* < 0.001). BA indirectly affected the behavior through behavior intention (BI), PBC indirectly affected the behavior through BI (*P* < 0.001), and SN had no significant direct effect on the BI, as shown in Fig. 2 (*P* > 0.05). Figures 3 and 4 present the results of multi-group SEM analyses for CEUS adoption behaviors of clinicians in leading and subordinate hospitals, respectively.

Table 3 shows the global fitting effect of multi-group SEM. The SEM analysis between groups forms consistency checks. Based on the results of fitting two groups of both leading and subordinate hospitals simultaneously, a baseline model without any restrictions could be obtained. The simulation results demonstrated that the model fit the data well, and the model form of the two groups was suitable for multiple group comparisons. As shown in Table 4, the significant impact of SN was detected on the clinicians’ behavior intention toward hepatic CEUS utilization in leading and subordinate hospitals (*P* < 0.001). SN had a positive effect on BI in leading hospitals, while they had a negative impact on BI in subordinate hospitals. Meanwhile, the correlation among SN, BA, and PBC was stronger in subordinate hospitals than those in the leading hospitals (*P* < 0.05). However, there were no significant differences between leading and subordinate hospitals in the path coefficients from BA to BI, from PBC to BI, or from BI to behavior (*P* > 0.05).

**Table 3:** Global fitting effect of multi-group structural equation model

model	CMIN	df	CFI	TLI	RMSEA	SRMR
Leading hospital	244.166	83	0.943	0.927	0.117	0.080
Subordinate hospital	195.893	83	0.941	0.925	0.097	0.065
Unconstrained	440.066	166	0.942	0.926	0.076	0.073
Measurement weights	454.915	176	0.941	0.929	0.074	0.077
Structural weights	472.753	180	0.938	0.927	0.075	0.079
Structural covariances	509.755	186	0.931	0.922	0.078	0.153
Structural residuals	511.704	188	0.931	0.923	0.077	0.156

**Table 4:** Comparison of all hypotheses and path coefficients

Hypotheses	Path	Leading hospital	Subordinate hospital	Critical value	*P* value
Physicians’ behavioral attitude influences their intention to use CEUS	BA → BI	0.324**	0.549*	1.080	0.280
Physicians’ subjective norm influences their intention to use CEUS	SN → BI	0.183*	-0.348*	3.721	< 0.001
Physicians’ perceived behavioral control influences their intention to use CEUS	PBC → BI	0.503**	0.716**	1.391	0.164
Physicians’ intention to use CEUS influences their utilization behavior	BI → Behavior	0.316**	0.298*	0.360	0.719
Physicians’ subjective norm and their attitude to use CEUS exist mutual relationship	SN←→BA	0.816**	0.857**	2.422	0.015
Physicians’ subjective norm and their perceived behavioral control exist mutual relationship	SN←→PBC	0.694**	0.823**	1.956	0.050
Physicians’ perceived behavioral control and their attitude to use CEUS exist mutual relationship	PBC←→BA	0.837**	0.891**	2.797	0.005

^*^P < 0.01, ^**^P < 0.001

## Discussion

Some commonly confirmed effective and appropriate health technologies are being implemented only in some higher-level institutions and far from widespread in lower-level hospitals [6], however, the mechanism underlying differences in clinicians’ adoption behavior at different levels of institutions within integrated care systems remained largely unknown. To address the research gap on health technology diffusion, this study utilized hepatic CEUS as a case example, formulated a hypothesis model based on TPB, and employed multi-group SEM to examine disparities in clinicians’ adoption behavior of CEUS and its influencing factors between leading and subordinate hospitals. The results showed that clinicians in the medical consortiums had a strong intention to adopt CEUS but scored lower in behavioral adoption. Significant differences were found between leading and subordinate hospitals in the influence of SN on BI. The correlation between SN, BA, and PBC was higher in subordinate hospitals. The findings revealed differences in the influencing mechanisms of clinicians’ technology adoption behaviors at different levels of institutions within the medical consortiums. It will not only directly guide the practice of promoting the utilization of CEUS but also provide clues for the expansion of other effective and appropriate technologies within the integrated care system.

It should be acknowledged that not all great intentions will inevitably result in targeted behavior. As shown in this study, clinicians within medical consortiums showed a strong intention to adopt hepatic CEUS, with scores of 4.113 and 4.376 for the leading and subordinate hospitals, respectively. Interestingly, however, the clinicians’ adoption behavior scores were lower, scoring 1.972 and 1.660 for leading and subordinate hospitals, respectively. This result indicated that clinicians at leading or subordinating hospitals had not yet widely applied hepatic CEUS technology in their clinical practice, even though they may have great intentions to do this. It suggested that the process of translating technology adoption intentions into actual technology utilization behavior is influenced by various factors, which require further investigation in subsequent research. For better translating intentions into actual behaviors, it is recommended that medical consortium managers build a more innovative and incentive environment and make some concrete and concerted efforts, which in turn translate clinicians’ adoption intention into the actual behavior of adopting innovative technologies widely confirmed as appropriate. Such findings were also in line with previous research [38], which revealed that there might be numerous barriers in the process of transforming technology adoption intention into actual technology utilization behavior [39]. It still highlights the importance and necessity of clarifying the influencing mechanism of clinicians’ adoption behavior, which would greatly benefit determining the demand and facilitators in the process of health technology adoption among leading and subordinate hospitals.

Consistent with previous research in the field of other health technology adoption [40, 41], this study found that clinicians’ attitudes toward hepatic CEUS and perceived behavioral control positively impacted their intentions to use it. Furthermore, this effect manifested consistently in leading hospitals and subordinate hospitals. It appeared that when clinicians recognized the value of adopting hepatic CEUS and perceived less resistance to its application in clinical practice, they would form favorable attitudes and perceived behavioral control, which in turn improved their intentions to adopt corresponding behaviors [42, 43]. Thus, it reminds us that more importance can be stressed on taking measures to make clinicians’ attitudes more positive toward regarding technologies and optimize their evaluation of the use.

Although this study confirmed that subjective norm was a reasonably good predictor of clinicians’ intention to provide hepatic CEUS, it is noteworthy that the effect of subjective norm on behavioral intentions was significantly different between leading hospitals and subordinate hospitals. As evidenced in this study, within leading hospitals, subjective norm positively influenced clinicians’ intention to utilize the appropriate technology, suggesting that clinicians generally improved their behavioral intentions when influential experts or other essential figures exerted social pressure on them [11]. This finding was in line with previous studies, which revealed that subjective norm had the most significant influence on nurses’ intention to implement patient safety behaviors [44]. Nevertheless, in subordinate hospitals, subjective norm was found to negatively affect clinicians’ intentions to use hepatic CEUS, which was contrary to previous studies [45–47]. One plausible explanation is the difference between new healthcare technology acceptance’s mandatory and voluntary contexts [23]. More specifically, clinicians in leading hospitals were more willing to receive new healthcare technology due to their functional positioning [48], while clinicians in subordinate hospitals were more passive in introducing new technologies because of the mandatory context of the medical consortiums. Clinicians in both leading and subordinate hospitals may be influenced by social pressure from their superiors. However, those in subordinate hospitals may be more resistant to adopting new health technologies, which can weaken their willingness to do so. Another possible reason is that clinicians in subordinate hospitals were influenced by their managers and the organizational environment to focus more on applying traditional, routine health technologies in their clinical practice [49]. As a result, their willingness to adopt new technologies is reduced even under the influence of social norms.

Furthermore, as demonstrated in this study, a significant correlation existed between behavioral attitudes, subjective norm, and perceived behavioral control, which was in accordance with TPB [9]. Even more remarkable, it revealed that the correlation between behavioral attitudes, subjective norm, and perceived behavioral control was significantly greater in subordinate hospitals than in the leading ones. Given the disparity in financial support and medical resources between leading and subordinate hospitals within Chinese medical consortiums, especially subordinate hospitals in resource-poor areas confronted with constraints of fewer organizational resources and lower individual capabilities [50], medical staff in subordinate hospitals had fewer types of health technologies to choose, which would probably make them rely more on specific technologies and also tend to develop common beliefs toward regarding technologies [51].

Based on research findings, several intervention strategies can be highlighted to further promote the diffusion and utilization of quality appropriate health technologies within the medical consortiums. First, member hospitals within the medical consortiums should improve support for health technology adoption behaviors by establishing information communication channels and improving feedback mechanisms, which will clarify needs and motivations in the health technology adoption process. Second, appropriate regulations should be formulated to promote the rational flow and allocation of patients, technology, and other resources within the medical consortiums. This is particularly crucial in ensuring the interests of subordinate hospitals throughout the operational process of the consortium, thereby harmonizing the relationship between leading and subordinate hospitals. Third, technical support should be further strengthened to promote vertical technology diffusion for subordinate hospitals. This will expand clinicians’ options regarding technologies, ultimately breaking their existing cognitive inertia and reinforcing their inclination toward introducing and adopting effective and appropriate new technologies in clinical practice.

The study focuses on the differences in influencing mechanisms of clinicians’ health technology adoption behavior between leading and subordinate hospitals within the medical consortiums. This will not only effectively bridge the research gap on health technology diffusion in integrated care systems, but also further clarify the theoretical mechanisms in the process of health technology diffusion. Moreover, the application of multi-group SEM not only made the influence of multiple factors on the outcome variables and their internal interactions simultaneously analyzed but also made it possible to investigate whether the paths in the mediation model were significantly different across leading and subordinate hospital groups.

Inevitably, there are still some limitations in this study that should be addressed in future research. Firstly, due to limited time and funds, we conducted a cross-sectional study and included only two provinces to carry out the survey. Future research may include more study sites and involve samples at different time points to form panel data, which will benefit the causality interpretation and make the results more robust. Secondly, since the study was based on TPB, some factors at the technological and organizational levels may be ignored. Further research could consider incorporating more relevant factors to ensure a comprehensive analysis. Finally, since there is no clearly identified sampling frame of clinicians using CEUS in China, the random sampling in this study from high and low hepatic cell carcinoma incidence regions has some bias in the representation of the sample. Furthermore, given that this study was conducted in regions with high and low hepatic cell carcinoma incidence, the regional limitation of the data prevents the conclusions from national generalization. Future studies could use a broader sample to compare results.

## Conclusion

This study performed multi-group SEM to investigate the differences in the influencing mechanism of clinicians’ adoption behavior for liver cancer screening technology between leading and subordinate hospitals within the medical consortium. It demonstrated that SN had a positive effect on BI in the leading hospitals, while they had a negative impact on BI in the subordinate hospitals. Moreover, the correlation among SN, BA and PBC was stronger in subordinate hospitals than those in leading hospitals. To further expand the application of technology, some practical supportive countermeasures are strongly recommended, such as building a better innovative and incentive environment, strengthening the technical support for subordinating hospitals and so on. The findings of this study will not only contribute to the existing knowledge on the field of technology adoption mechanisms among different level institutions but also facilitate the dissemination and utilization of effective and appropriate innovative technologies within the integrated care system.

### Electronic supplementary material

Below is the link to the electronic supplementary material.

Supplementary Material 1

Supplementary Material 2

Supplementary Material 3

Supplementary Material 4

## Data Availability

The datasets generated and/or analyzed during the current study are available from the corresponding author on reasonable request.

## Abbreviations

CEUS: Contrast-enhanced ultrasound technology
TPB: Theory of planned behavior
SEM: Structural equation modeling
BA: Behavioral attitudes
SN: Subjective norm
PBC: Perceived behavioral control
CFI: Comparative fit index
TLI: Tucker-Lewis Index
RMSEA: Root mean square error of approximation
SRMR: Standardized root mean square residual
KMO: Kaiser-Meyer-Olkin
AVE: Average variance extracted
CR: Composite reliability
