# Supplementary material for: Differences in influencing mechanism of clinicians’ adoption behavior for liver cancer screening technology between the leading and subordinate hospitals within medical consortiums
Source: BMC Cancer. 2024 Apr 23;24:514. doi: 10.1186/s12885-024-12281-y (PMC11040858; doi:10.1186/s12885-024-12281-y)
Supplement: Supplementary file 1 — Supplementary Material 1 [file 12885_2024_12281_MOESM1_ESM.doc]

**Figure** Sampling flow chart
