# Supplementary material for: Differences in influencing mechanism of clinicians’ adoption behavior for liver cancer screening technology between the leading and subordinate hospitals within medical consortiums
Source: BMC Cancer. 2024 Apr 23;24:514. doi: 10.1186/s12885-024-12281-y (PMC11040858; doi:10.1186/s12885-024-12281-y)
Supplement: Supplementary file 2 — Supplementary Material 2 [file 12885_2024_12281_MOESM2_ESM.doc]

**Research questionnaire of CEUS utilization**

**among physicians in China**

**Part 1. Personal information card**

1. Please choose your gender.

A. Male B. Female

2. Please write down your age: ________

3. Please choose your educational Level.

A. Junior college or below B. Bachelor C. Master D. Doctor

4. Please choose your professional Title.

A. Junior B. Intermediate C. Senior

5. Please choose your years in practice.

A. <5 years B. 5~10 years C. 11~20years D. 21~30 years E. >30 years

6. Please choose the level of medical institution you are in.

A. Leading hospital B. Subordinate hospital

**Part 2. Behavior to use contrast-enhanced ultrasound (CEUS)**

Notes. There are 6 numbers (0, 1, 2, 3, 4, 5) on the right side of each item, where “0” means “never”, “1” means “Very low (0-20%]”, “2” means “Low (20%-40%]”, “3” means “Medium (40%-60%]”, “4” means “High (60%-80%]”, and “5” means “Very high (80%-100%]” . Please tick or circle the number that best fits your real feelings on the item.

| Over the past year, the probability of my recommending a referral of a suspicious patient to a superior hospital with the technical ability to apply CEUS under appropriate clinical situations. | 0 | 1 | 2 | 3 | 4 | 5 |
| --- | --- | --- | --- | --- | --- | --- |
| Over the past year, the probability of my ordering a hepatic CEUS on all working days. | 0 | 1 | 2 | 3 | 4 | 5 |
| Over the past year, the probability of my advising my colleagues to use CEUS in the early diagnosis of liver cancer on a larger scale. | 0 | 1 | 2 | 3 | 4 | 5 |

**Part 3. The scale of Theory of Planned Behaviour**

Notes. There are 5 numbers (1, 2, 3, 4, 5) on the right side of each item, where “1” means “Strongly disagree”, “2” means “Disagree”, “3” means “Neutral”, “4” means “Agree”, and “5” means “Strongly agree”. Please tick or circle the number that best fits your real feelings on the item.

| ***Behavior intention*** | | | | | |
| --- | --- | --- | --- | --- | --- |
| If there is an opportunity, I would like to apply contrast-enhanced ultrasound to the early diagnosis of liver cancer. | 1 | 2 | 3 | 4 | 5 |
| I would like to learn from my peers the skills and experience of using contrast-enhanced ultrasound in the diagnosis of early liver cancer. | 1 | 2 | 3 | 4 | 5 |
| I would like to recommend the use of contrast-enhanced ultrasound to diagnose early liver cancer to the surrounding doctors. | 1 | 2 | 3 | 4 | 5 |
| ***Behavior attitude*** | | | | | |
| I think it’s a right thing to use CEUS for early diagnosis of liver cancer. | 1 | 2 | 3 | 4 | 5 |
| I think it’s a wise choice to use CEUS for early diagnosis of liver cancer. | 1 | 2 | 3 | 4 | 5 |
| I think it’s good for all to use CEUS for early diagnosis of liver cancer. | 1 | 2 | 3 | 4 | 5 |
| ***Subjective norm*** | | | | | |
| People who are important to me tend to use CEUS for early diagnosis of liver cancer. | 1 | 2 | 3 | 4 | 5 |
| People who are important to me have a positive attitude to use CEUS for early diagnosis of liver cancer. | 1 | 2 | 3 | 4 | 5 |
| People who are important to me think it’s a right thing to use CEUS for early diagnosis of liver cancer. | 1 | 2 | 3 | 4 | 5 |
| ***Perceived behavioral control*** | | | | | |
| Using CEUS can make me have more choice in diagnosing liver cancer. | 1 | 2 | 3 | 4 | 5 |
| Using CEUS can increase my confidence in diagnosing liver cancer. | 1 | 2 | 3 | 4 | 5 |
| Using CEUS can make my diagnosis more recognized. | 1 | 2 | 3 | 4 | 5 |
