# Supplementary material for: Differences in influencing mechanism of clinicians’ adoption behavior for liver cancer screening technology between the leading and subordinate hospitals within medical consortiums
Source: BMC Cancer. 2024 Apr 23;24:514. doi: 10.1186/s12885-024-12281-y (PMC11040858; doi:10.1186/s12885-024-12281-y)
Supplement: Supplementary file 3 — Supplementary Material 3 [file 12885_2024_12281_MOESM3_ESM.doc]

**Table A** Reliability and convergent validity

| Constructs | Items | Factor Loadings | Cronbach’s  Alpha | AVE | CR |
| --- | --- | --- | --- | --- | --- |
| Behavior | ①Over the past year, the probability of my recommending a referral of a suspicious patient to a superior hospital with the technical ability to apply CEUS under appropriate clinical situations | 0.586 | 0.827 | 0.653 | 0.844 |
|  | ②Over the past year, the probability of my ordering a hepatic CEUS on all working days | 0.838 |  |  |  |
|  | ③Over the past year, the probability of my advising my colleagues to use CEUS in the early diagnosis of liver cancer on a larger scale | 0.922 |  |  |  |
| Behavior intention | ①If there is an opportunity, I would like to apply contrast-enhanced ultrasound to the early diagnosis of liver cancer | 0.956 | 0.948 | 0.858 | 0.948 |
|  | ②I would like to learn from my peers the skills and experience of using contrast-enhanced ultrasound in the diagnosis of early liver cancer | 0.890 |  |  |  |
|  | ③I would like to recommend the use of contrast-enhanced ultrasound to diagnose early liver cancer to the surrounding doctors | 0.931 |  |  |  |
| Behavior attitude | ① I think it's a right thing to use CEUS for early diagnosis of liver cancer | 0.869 | 0.917 | 0.788 | 0.918 |
|  | ②I think it's a wise choice to use CEUS for early diagnosis of liver cancer | 0.919 |  |  |  |
|  | ③I think it's good for all to use CEUS for early diagnosis of liver cancer | 0.875 |  |  |  |
| Subjective norm | ①People who are important to me tend to use CEUS for early diagnosis of liver cancer | 0.881 | 0.933 | 0.825 | 0.934 |
|  | ②People who are important to me have a positive attitude on using CEUS for early diagnosis of liver cancer | 0.916 |  |  |  |
|  | ③People who are important to me think it’s a right thing to use CEUS for early diagnosis of liver cancer | 0.927 |  |  |  |
| Perceived behavior control | ①Using CEUS can make me have more choice in diagnosing liver cancer | 0.899 | 0.935 | 0.830 | 0.936 |
|  | ②Using CEUS can increase my confidence in diagnosing liver cancer | 0.913 |  |  |  |
|  | ③Using CEUS can make my diagnosis more recognized | 0.921 |  |  |  |

**Table B1** Rotated factor matrix

|  | Factor 1 | Factor 2 | Factor 3 | Factor 4 | Factor 5 |
| --- | --- | --- | --- | --- | --- |
| Behavior 1 |  |  | 0.911 |  |  |
| Behavior 2 |  |  | 0.939 |  |  |
| Behavior 3 |  |  | 0.897 |  |  |
| BI 1 |  |  |  |  | 0.842 |
| BI 2 |  |  |  |  | 0.829 |
| BI 3 |  |  |  |  | 0.821 |
| BA 1 |  |  |  | 0.740 |  |
| BA 2 | 0.562 |  |  | 0.614 |  |
| BA 3 |  |  |  | 0.680 |  |
| SN 1 |  | 0.787 |  |  |  |
| SN 2 |  | 0.837 |  |  |  |
| SN 3 |  | 0.796 |  |  |  |
| PBC 1 | 0.864 |  |  |  |  |
| PBC 2 | 0.811 |  |  |  |  |
| PBC 3 | 0.865 |  |  |  |  |

Abbreviations: BI, Behavior intention; BA, Behavior attitude; SN, Subjective norm; PBC, Perceived behavior control.

**Table B2** Total variance explanation

| Factor | Variance (%) | Cumulative percentage |
| --- | --- | --- |
| Factor 1 | 18.76 | 18.76 |
| Factor 2 | 17.96 | 36.72 |
| Factor 3 | 18.04 | 54.76 |
| Factor 4 | 13.93 | 68.69 |
| Factor 5 | 16.55 | 85.24 |

Notes: Factor 1 “perceived behavior control”, Factor 2 “subjective norm”, Factor 3 “behavior”, Factor 4 “behavior attitude”, and Factor 5 “behavior intention”.
