# Supplementary material for: Differences in influencing mechanism of clinicians’ adoption behavior for liver cancer screening technology between the leading and subordinate hospitals within medical consortiums
Source: BMC Cancer. 2024 Apr 23;24:514. doi: 10.1186/s12885-024-12281-y (PMC11040858; doi:10.1186/s12885-024-12281-y)
Supplement: Supplementary file 4 — Supplementary Material 4 [file 12885_2024_12281_MOESM4_ESM.doc]

**Table** Comparison in each item between early respondents and late respondents

| Variables | Categories | Early respondents  N= 29 | Late respondents  N= 29 | p-value ^a^ |
| --- | --- | --- | --- | --- |
| Gender | Male | 18 (62.1%) | 18 (62.1%) | 1.000^b^ |
|  | Female | 11 (37.9%) | 11 (37.9%) |  |
| Age | <35 years old | 12 (41.4%) | 10 (34.5%) | 0.626^c^ |
|  | 35~ 44 years old | 12 (41.4%) | 16 (55.2%) |  |
|  | >45 years old | 5 (17.2%) | 3 (10.3%) |  |
| Education level | Junior college or below | 0 (0.0%) | 1 (3.4%) | 0.429^c^ |
|  | Bachelor | 14 (48.3%) | 17 (58.6%) |  |
|  | Master or above | 15 (51.7%) | 11 (37.9%) |  |
| Professional title | Junior | 15 (51.7%) | 8 (27.6%) | 0.136^b^ |
|  | Intermediate | 7 (24.1%) | 13 (44.8%) |  |
|  | Senior | 7 (24.1%) | 8 (27.6%) |  |
| Years in practice | ＜5 years | 8 (27.6%) | 6 (20.7%) | 0.455^c^ |
|  | 5~10 years | 11 (37.9%) | 8 (27.6%) |  |
|  | 11~20 years | 6 (20.7%) | 12 (41.4%) |  |
|  | 21~30 years | 3 (10.3%) | 3 (10.3%) |  |
|  | ＞30 years | 1 (3.4%) | 0 (0.0%) |  |
| Behavior score mean (SD) |  | 1.8 (1.0) | 1.7 (1.2) | 0.752^d^ |
| Behavioral intention score mean (SD) |  | 4.4 (0.7) | 4.4 (0.7) | 0.997^d^ |
| Subjective norm score mean (SD) |  | 4.2 (1.0) | 4.2 (0.8) | 0.891^d^ |
| Behavioral attitude score mean (SD) |  | 4.4 (0.8) | 4.2 (0.8) | 0.335^d^ |
| Perceived behavioral control score mean (SD) |  | 4.4 (0.7) | 4.4 (0.8) | 0.775^d^ |

^a^ Statistically significant at α < 0.05. ^b^ Chi-square test. ^c^ Fisher’s exact test. ^d^ Paired-samples T test.
